# Vitamin D Status in Children With Short Stature: Accurate Determination of Serum Vitamin D Components Using High-Performance Liquid Chromatography–Tandem Mass Spectrometry

**DOI:** 10.3389/fendo.2021.707283

**Published:** 2021-10-13

**Authors:** Bei Xu, Yue Feng, Lingling Gan, Yamei Zhang, Wenqiang Jiang, Jiafu Feng, Lin Yu

**Affiliations:** Department of Clinical Laboratory, Mianyang Central Hospital, School of Medicine, University of Electronic Science and Technology of China, Mianyang, China

**Keywords:** short stature, 25-hydroxyvitamin D2, 25-hydroxyvitamin D3, 3-epi-25(OH)D3, liquid chromatography–tandem mass spectrometry (LC-MS/MS)

## Abstract

**Objective:**

Vitamin D is critical for calcium and bone metabolism. Vitamin D insufficiency impairs skeletal mineralization and bone growth rate during childhood, thus affecting height and health. Vitamin D status in children with short stature is sparsely reported. The purpose of the current study was to investigate various vitamin D components by high-performance liquid chromatography–tandem mass spectrometry (LC-MS/MS) to better explore vitamin D storage of short-stature children *in vivo*.

**Methods:**

Serum circulating levels of 25-hydroxyvitamin D2 [25(OH)D2], 25-hydroxyvitamin D3 [25(OH)D3], and 3-epi-25-hydroxyvitamin D3 [3-epi-25(OH)D3, C3-epi] were accurately computed using the LC-MS/MS method. Total 25(OH)D [t-25(OH)D] and ratios of 25(OH)D2/25(OH)D3 and C3-epi/25(OH)D3 were then respectively calculated. Free 25(OH)D [f-25(OH)D] was also measured.

**Results:**

25(OH)D3 and f-25(OH)D levels in short-stature subgroups 2 (school age: 7~12 years old) and 3 (adolescence: 13~18 years old) were significantly lower compared with those of healthy controls. By contrast, C3-epi levels and C3-epi/25(OH)D3 ratios in all the three short-stature subgroups were markedly higher than the corresponding healthy cases. Based on cutoff values developed by Endocrine Society Recommendation (but not suitable for methods 2 and 3), sufficient storage capacities of vitamin D in short-stature subgroups 1, 2, and 3 were 42.8%, 23.8%, and 9.0% as determined by Method 3 [25(OH)D2/3+25(OH)D3], which were lower than those of 57.1%, 28.6%, and 18.2% as determined by Method 1 [25(OH)D2+25(OH)D3+C3-epi] and 45.7%, 28.5%, and 13.6% as determined by Method 2 [25(OH)D2/3+25(OH)D3+C3-epi]. Levels of 25(OH)D2 were found to be weakly negatively correlated with those of 25(OH)D3, and higher 25(OH)D3 levels were positively correlated with higher levels of C3-epi in both short-stature and healthy control cohorts. Furthermore, f-25(OH)D levels were positively associated with 25(OH)D3 and C3-epi levels in children.

**Conclusions:**

The current LC-MS/MS technique can not only separate 25(OH)D2 from 25(OH)D3 but also distinguish C3-epi from 25(OH)D3. Measurement of t-25(OH)D [25(OH)D2+25(OH)D3] alone may overestimate vitamin D storage in children, and short-stature children had lower vitamin D levels compared with healthy subjects. Ratios of C3-epi/25(OH)D3 and 25(OH)D2/25(OH)D3 might be alternative markers for vitamin D catabolism/storage in short-stature children. Further studies are needed to explore the relationships and physiological roles of various vitamin D metabolites.

## Introduction

Short stature is a global public health problem (1). It is defined statistically as height less than 2 standard deviations (SD) of age- and sex-matched population (1, 2). Short stature with general health can lead to several physical or psychological concerns in modern society. Severe short stature is vulnerably linked with diverse developmental, educational, and social problems especially for children (3).

Stature is hereditary trait regulated by both genetic and environmental factors. Manipulation of environmental factors may be an effective strategy to maximize the growth potential of children (4). Short stature is associated with various underlying environmental factors, including inadequate dietary intake (4), essential nutrition or trace element deficiency (5), and exposure to environmental pollutants (6). Vitamin D plays essential roles in function and maintenance of bone health by regulating calcium and phosphate homeostasis throughout life (7). Previous studies established that vitamin D deficiency reduces skeletal mineralization and bone growth rate (8). Infants and young children are special risk groups of vitamin D deficiency due to their rapid growth with high nutritional requirements. However, there is paucity of data on levels of vitamin D status in short-stature children. Therefore, there is a need to explore the relationships between vitamin D status and patients with short stature.

Circulating 25-hydroxyvitamin D [25(OH)D] is currently widely used as a functional indicator for vitamin D status (9), which mainly comprises two biologically inactive precursors including 25-hydroxyvitamin D2 [25(OH)D2] and 25-hydroxyvitamin D3 [25(OH)D3] (10, 11). 25(OH)D2 is mainly sourced from plants and only enters body *via* diet, whereas 25(OH)D3 is endogenously synthesized in skin *via* UV irradiation of 7-dehydrocholesterol (12). Several studies had considered that 25(OH)D2 is as effective as 25(OH)D3 in improving bone health (13), while others averred that 25(OH)D3 is more potent than 25(OH)D2 in maintaining 25(OH)D levels, with a differential potency of at least 3-fold (14). Both 25(OH)D2 and 25(OH)D3 should be tested simultaneously to comprehensively assess vitamin D status. So far, few studies have quantified both 25(OH)D2 and 25(OH)D3 levels in children with short stature. Thus, there is urgent need to explore the internal relationships between 25(OH)D2 and 25(OH)D3 status and short stature disease.

Recent studies reported that vitamin D3 metabolites are further metabolized through the C3-epimerization pathway (15). 25(OH)D3 undergoes epimerization in the liver to produce 3-epi-25-hydroxyvitamin D3 [3-epi-25(OH)D3, C3-epi] (the hydroxyl group in the C-3 position of A-ring changes from α to β orientation) (16). Although the physiological role of C3-epi is still obscure, previous studies have reported elevated C3-epi proportions in mothers and newborns, indicating importance of epimers in pregnancy and early development (17, 18). However, C3-epi presumably does not function as a storage pool because 3-epimerization is irreversible. Some studies showed that C3-epi has weaker calcemic regulatory effects compared with its non-epimeric form (19). It induces phospholipid synthesis in pulmonary alveolar type II cells and suppresses parathyroid hormone secretion with comparable amounts with non-epimeric metabolites (20, 21). Conversely, its conversion product in kidneys, namely, 3-epi-1α,25(OH)2D3, performs stronger differentiation or anti-proliferative activities than non-epimeric compounds *in vitro* (22). Additionally, it has greater metabolic stability compared with 1α,25(OH)2D3 despite having inequivalent binding strength to vitamin D receptors (VDR). This allows 3-epi-1α,25(OH)2D3 to remain in free form and hence participate in physiological processes (22). Numerous studies have found that the identical molecular weight and molecular physical–chemical property of C3-epi may lead to inaccuracies in 25(OH)D3 measurements. These findings render the necessity for specific separate detection of 25(OH)D3 and C3-epi (23). Liquid chromatography–tandem mass spectrometry (LC-MS/MS) technology can quantify 25(OH)D2 and 25(OH)D3 and distinguish C3-epi from 25(OH)D3 simultaneously. The purpose of the current study was to explore levels of 25(OH)D2, 25(OH)D3, and C3-epi in short-stature children using the LC-MS/MS method. Values of total 25(OH)D [t-25(OH)D] as well as ratios of C3-epi/25(OH)D3 and 25(OH)D2/25(OH)D3 were then computed. The results of the current study are expected to provide a scientific bearing for the diagnosis, treatment, and prognosis evaluation of children with short stature.

## Participants and Methods

### Study Participants

The current study recruited patients who visited the child healthcare department for short-stature problems between January 2017 and January 2021 in Mianyang Central Hospital, Sichuan Province, China, as study participants. Clinically, individual diagnostic categories are often indistinguishable, and the demarcation of diagnoses often leads to joint diagnoses. Therefore, the current study summarized all subtypes under general term and boundary definition of short stature but excluded those caused by genetic, syndromic, organic, and psychosocial conditions.

The diagnosis of short stature was based on a previous diagnostic guideline (24). The height of the children was determined in relevance to their age, health status, family, and history of development. Their physical parameters (weight, height of sitting posture) and external signs of genetic conditions were also recorded. The bone age of each child was determined from X-ray images of the hands and wrists. Those with heights exceeding two standard deviations (SD) below average height of the corresponding gender and age, as stipulated in the standards for Chinese children and adolescents, were included in the study.

Exclusion criteria included children with other conditions such as growth hormone deficiency, multiple pituitary hormone deficiency, hypothyroidism, skeletal development disorder, intracranial tumor, chromosomal disease, chronic systemic disease, familial short stature, physical puberty delay, severe malnutrition, and other known causes of short stature. Participants who had received growth hormone, gonadotropin releasing hormone, or antihypertensive treatment were also excluded by a qualified pediatrician.

Healthy participants were assigned into the control group. The current study was approved by the Medical Ethics Committee of Mianyang Central Hospital.

### Collection of Blood Samples

Venous blood was collected between 6:00 and 10:00 a.m. after overnight fasting to eliminate the influence of diet on serum measurements. The blood was centrifuged at 3,000 rpm for 15 min to obtain serum.

### Determination of 25(OH)D2, 25(OH)D3, and 3-epi-25(OH)D3 by LC-MS/MS

This was undertaken based on our previously described study (25). Briefly, 10 μl of mixed internal standard was added to 200 μl of serum samples and then mixed with 1,000 μl of extraction solution (tert-butyl methyl ether). The supernatant was collected after vortex and centrifugation. Resulting solutions were dried under nitrogen gas and redissolved in 125 μl of methanol with 0.1% formic acid. The mixture was then vortex-mixed and centrifuged at 13,000 rpm for 5 min, and the resulting supernatant was transferred to a 96-well sample plate, which was then sealed and transferred to an autosampler. Calibrators and quality controls were prepared based on the same procedure.

Chromatographic analysis was performed on a Shimadzu LC-30AD UHPLC system equipped with a Kinetex 2.6 μm C8 100A column. Mobile phase A consisted of water with 0.1% acetic acid, and mobile phase B consisted of methanol with 0.1% acetic acid. Fifteen microliters of the sample solutions was injected into the LC system using a column temperature of 45°C and a flow rate of 0.6 ml/min. Mass spectrometer detection and quantification were undertaken in positive mode using multiple reaction monitoring (MRM) mode. Optimized parameters for mass detection were as follows: curtain gas was 35 psi; temperature was 550°C; ion spray voltage was 5,500 V; gas 1 and gas 2 (nitrogen) were both set at 60 psi; and the dwell time was 100 ms. Analyst^®^ MD software (version number: 1.6.3) was used for chromatogram output, and MultiQuant™ MD software (version number: 3.0.2) was performed for data processing.

### Detection of f-25(OH)D

The free 25(OH)D ELISA kit was obtained from DIAsource ImmunoAssays SA (Belgium) to detect f-25(OH)D levels. The assay was calibrated against Rate Dialysis, which is the gold standard method for the determination of free hormones. Final concentrations were analyzed using the RT-6100 enzyme label analyzer (Redu Life Science Co., Ltd., Shenzhen, China) based on kit instructions.

### Evaluation of Vitamin D Nutritional Status

The capacity of vitamin D3 to store vitamin D is two to three times higher compared with that of vitamin D2. To provide alternative methods for accurate or sufficient vitamin D storage converted into active vitamin D [1,25(OH)_2_D], three different computation methods were applied, including 25(OH)D2+25(OH)D3+C3-epi (Method 1), 25(OH)D2/3+25(OH)D3+C3-epi (Method 2), and 25(OH)D2/3+25(OH)D3 (Method 3) (25). Method 1 is most widely applied to determine t-25(OH)D values using various immunological assays, whereas Methods 2 and 3 may better represent vitamin D storage converted into 1,25(OH)_2_D. Method 3 is considered more suitable for the determination of the active status of 25(OH)D in circulation after removal of C3-epi from 25(OH)D3. Recommendations of Endocrine Society aver that a 25(OH)D concentration of <20 ng/ml is indicative of vitamin D deficiency, whereas a concentration in the range of 21–29 ng/ml indicates insufficiency. In addition, a concentration of >30 ng/ml is considered sufficient (11).

### Statistical Analyses

Statistical analyses were performed using SPSS 25.0 software (International Business Machines Corp., USA). Data were expressed as mean ± standard deviation (SD) for normally distributed continuous data and analyzed using Student’s t-test between two study groups. The M=median and interquartile range (IQR) were selected for non-normally distributed variables and analyzed by Mann–Whitney U tests. One-way ANOVA was used to analyze differences between means of more than two groups for equal variances. Welch’s approximate analysis was used followed by Dunnett’s T3 test if the variances are uneven. The strength of the relationship between selected metabolite parameters and commonly used fasting lipid profiles was determined using Pearson or Spearman bivariate correlation analysis for normal or skew distribution. p < 0.05 was considered statistically significant.

## Results

### General Characteristics of Participants

A total of 99 eligible short-stature children aged between 1 and 18 years, including 45 males and 54 females, were recruited in the current study. In addition, 186 healthy participants were assigned to the control group, among whom were 86 males and 100 females. Influence of age on outcomes was minimized by grouping children into three subgroups: Subgroup 1 (preschool age) aged between 1 and 6 years; Subgroup 2 (school age) aged between 7 and 12 years; and Subgroup 3 (adolescence) aged between 13 and 18 years. **Table 1** shows the basic clinical characteristics of participants. The mean height SDS of the short-stature group was -2.87 ± 0.34. Patients with short stature had significantly lower height and weight compared with healthy controls (both *p* < 0.001). No significant differences in age (t = 1.17, p = 0.367), sex (χ2 = 0.25, p = 0.426), and BMI (t = 1.173, p = 0.242) were observed between short-stature children and healthy controls. Furthermore, biochemical indices including calcium (Ca), phosphate (PHOS), free triiodothyronine (FT3), free thyroxine (FT4), thyroid-stimulating hormone (HTSH), parathyroid hormone (PTH), and alkaline phosphatase (ALP), which are associated with children growth and development, were also not statistically significant between the two study groups.

**Table 1:** General characteristics of the study cohort.

Items	Healthy control (n = 186)	Short stature (n = 99)	p values
Sex (male/female)	86/100	45/54	*0.426*
Age (years)	8.5 ± 2.5	8.3 ± 1.9	*0.367*
Height (cm)	139.32 ± 17.03	125.84 ± 20.04	*<0.001*
Height SDS	0.5 (-0.2, 1.0)	-2.87 (-2.7, -3.1)	*<0.001*
Weight (kg)	34.54 ± 11.17	29.03 ± 11.19	*<0.001*
Weight SDS	0.65 ± 0.21	-0.81 ± 0.24	*<0.001*
BMI (kg/m^2^)	17.20 ± 1.87	17.48 ± 1.69	*0.242*
BMI SDS	0.20 (-0.19, 0.73)	0.31 (-0.12, 0.68)	*0.196*
Ca (mmol/L)	2.51 ± 0.13	2.52 ± 0.10	*0.662*
PHOS (mmol/L)	1.66 ± 0.21	1.68 ± 0.16	*0.659*
FT3 (pg/mL)	3.69 ± 0.48	3.95 ± 0.46	*0.331*
FT4 (ng/dL)	1.10 ± 0.11	0.99 ± 0.22	*0.439*
HTSH (μIU/mL)	2.30 ± 1.19	2.57 ± 1.51	*0.062*
PTH (pg/mL)	32.56 ± 17.92	36.87 ± 18.72	*0.693*
ALP (U/L)	254.52 ± 78.15	249.81 ± 76.29	*0.591*

Ca, calcium; PHOS, phosphate; FT3, free triiodothyronine; FT4, free thyroxine; HTSH, thyroid-stimulating hormone; PTH, parathyroid hormone; ALP, alkaline phosphatase.

Data were expressed by mean ± SD, or median (P25, P75).

The p value determines statistical significance between the two compared groups. P<0.001 was considered statistically significant.

### Levels of Serum Vitamin D Components

**Figure 1:**
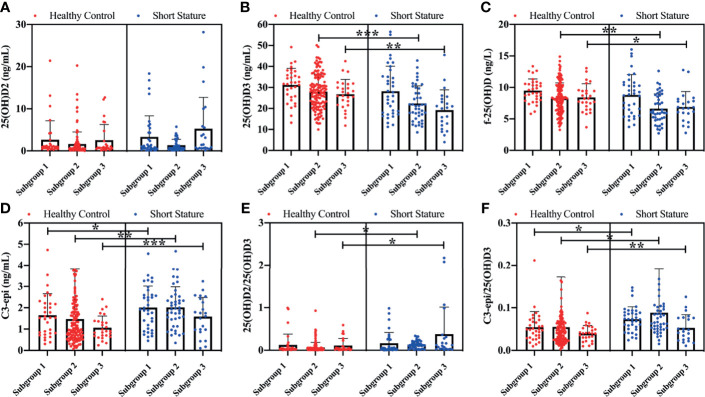
Comparison of various vitamin D components between short stature and healthy control subgroups. Levels of 25(OH)D2 **(A)**, 25(OH)D3 **(B)**, f-25(OH)D **(C)**, and C3-epi **(D)**, and ratios of 25(OH)D2/25(OH)D3 **(E)** and C3-epi/25(OH)D3 **(F)** for all subgroups. *P < 0.05, **P < 0.01, and ***P < 0.001 were considered statistically significant. 1) Subgroup 1 (preschool age): 1~6 years old; 2) Subgroup 2 (school age): 7~12 years old; and 3) Subgroup 3 (adolescence): 13~18 years old.

Findings of the current study showed that both serum levels of 25(OH)D3 (t = 3.825, p < 0.001; t = 3.121, p = 0.003) and f-25(OH)D (t = 3.848, p = 0.002; t = 2.282, p = 0.017) in subgroups 2 and 3 of short-stature patients were significantly lower compared with those of healthy controls, whereas C3-epi levels (z = 2.548, p = 0.023; z = 3.282, z = 0.007; z = 4.848, p < 0.001) and C3-epi/25(OH)D3 ratios (z = 2.845, p = 0.022; z = 2.285, z = 0.027; z = 3.788, p = 0.002) were all markedly higher in subgroups 1, 2, and 3 than those of healthy participants for the corresponding control subgroups (**Figure 1**). We further compared levels of serum vitamin D components among different subgroups. Findings showed significant statistical differences in circulating 25(OH)D3 (F = 35.63, p < 0.001), C3-epi (H = 28.62, p < 0.001), and f-25(OH)D (F = 31.25, p < 0.001) levels as well as C3-epi/25(OH)D3 ratios (H = 19.65, p < 0.001) among various subgroups. Generally, increase in age correlated with decrease in all the studied indicators.

### Evaluation of Vitamin D Nutritional Status

Percentages of vitamin D status among subgroups in short-stature and healthy participants are presented in **Table 2**. Specifically, sufficient storage capacities of vitamin D in short-stature subgroups were only 42.8%, 23.8%, and 9.0% as determined by Method 3, which were lower compared with those of 57.1%, 28.6%, and 18.2% as determined by Method 1 and 45.7%, 28.5%, and 13.6% as determined by Method 2. Notably, the current universally accepted clinical cutoffs (developed by Endocrine Society Recommendation) for vitamin D status were established using immunoassays, which are incapable of isolating C3-epi. Therefore, they are not suitable to evaluate the vitamin D status using Methods 2 and 3, which are likely to overestimate proportions of vitamin D deficiency and insufficiency. The current study just provided two alternative methods here to represent accurate or sufficient vitamin D storage converted into active vitamin D [1,25(OH)_2_D]. Appropriate cutoff values for these forms of vitamin D need to be defined further, given the lack of consensus on adequate levels of vitamin D.

**Table 2:** Evaluation of the subject’s vitamin D nutritional status [%(case/total)].

Subjects		Healthy Control			Short Stature		
		Subgroup 1	Subgroup 2	Subgroup 3	Subgroup 1	Subgroup 2	Subgroup 3
	Deficiency	0 (0/32)	10.9 (14/128)	3.8 (1/26)	14.3 (5/35)	35.7 (15/42)	22.7 (5/22)
**Methods 1**	Insufficiency	25.0 (8/32)	34.4 (44/128)	46.2 (12/26)	28.6 (10/35)	35.7 (15/42)	59.1 (13/22)
	Sufficiency	75.0 (24/32)	54.7 (70/128)	50.0 (13/26)	57.1 (20/35)	28.6 (12/42)	18.2 (4/22)
	Deficiency	3.1 (1/32)	13.3 (17/128)	15.4 (4/26)	22.9 (8/35)	40.5 (17/42)	45.5 (10/22)
**Methods 2**	Insufficiency	25.0 (8/32)	38.3 (49/128)	46.2 (12/26)	31.4 (11/35)	31.0 (13/42)	40.9 (9/22)
	Sufficiency	71.9 (23/32)	48.4 (62/128)	38.4 (10/26)	45.7 (16/35)	28.5 (12/42)	13.6 (3/22)
	Deficiency	3.1 (1/32)	14.9 (19/128)	15.4 (4/26)	28.6 (10/35)	42.9 (18/42)	45.5 (10/22)
**Methods 3**	Insufficiency	34.4 (11/32)	45.3 (58/128)	50.0 (13/26)	28.6 (10/35)	33.3 (14/42)	45.5 (10/22)
	Sufficiency	62.5 (20/32)	39.8 (51/128)	34.6 (9/26)	42.8 (15/35)	23.8 (10/42)	9.0 (2/22)

Method 1 = 25(OH)D2+25(OH)D3+C3-epi. Method 2 = 25(OH)D2/3 + 25(OH)D3+C3-epi. Method 3 = 25(OH)D2/3 + 25(OH)D3.

Vitamin D storage in all subgroups was determined, and results are presented in **Table 3**. Short-stature children in subgroup 2 had significantly lower vitamin D levels (t = 4.575, p < 0.001; t = 4.212, p < 0.001; t = 2.861, p = 0.005) compared with healthy children as determined by all the three methods, whereas vitamin D levels in short-stature children in subgroup 3 only significantly decreased in comparison to healthy participants as determined by Methods 1 and 3 (t = 2.568, p = 0.007; t = 6.115, p < 0.001). Moreover, children in subgroup 1 had markedly higher vitamin D levels compared with those in subgroups 2 and 3 as determined by the three methods irrespective of short-stature subgroups (Subgroups 1 *vs.* 2: t = 6.313, p < 0.001; t = 6.103, p < 0.001; t = 5.564, p < 0.001. Subgroups 1 *vs.* 3: t = 5.896, p < 0.001; t = 6.589, p < 0.001; t = 6.352, p < 0.001) or groups of healthy children (Subgroups 1 *vs.* 2: t = 2.561, p = 0.012; t = 2.458, p = 0.020; t = 2.313, p = 0.020. Subgroups 1 *vs.* 3: t = 3.131, p = 0.004; t = 3.125, p = 0.005; t = 3.025, p = 0.004.). However, there were no statistically significant differences in vitamin D levels between subgroups 2 and 3.

**Table 3:** Evaluation of vitamin D storage in subjects.

Subjects	Healthy control			Short stature			F, p value
	Subgroup 1	Subgroup 2	Subgroup 3	Subgroup 1	Subgroup 2	Subgroup 3	
VitD storage by Method 1 (ng/mL)	35.45 ± 6.91	31.14 ± 9.15^1^	30.39 ± 7.45^2^	33.08 ± 12.27	24.76 ± 8.89^3,^ ^a^	25.06 ± 9.24^4,^ ^b^	7.316, *p<0.001*
VitD storage by Method 2 (ng/mL)	33.70 ± 7.58	30.05 ± 8.87^1^	28.69 ± 7.11^2^	30.85 ± 12.34	23.84 ± 8.83^3,^ ^a^	21.55 ± 9.10^4^	7.964, *p<0.001*
VitD storage by Method 3 (ng/mL)	32.05 ± 7.15	28.58 ± 8.34^1^	27.63 ± 6.89^2^	29.30 ± 11.60	22.81 ± 8.39^3,^ ^a^	20.98 ± 8.74^4,^ ^b^	7.517, *p<0.001*

a Compared with healthy children in subgroup 2, p < 0.05.

b Compared with healthy children in subgroup 3, p < 0.05.

^1,2^Compared with children in subgroup 1 in the healthy cohort, p < 0.05.

^3,4^Compared with children in subgroup 1 in the short stature group, p<0.05.

### Associations of Serum 25(OH)D2, 25(OH)D3, and t-25(OH)D Levels

**Figure 2:**
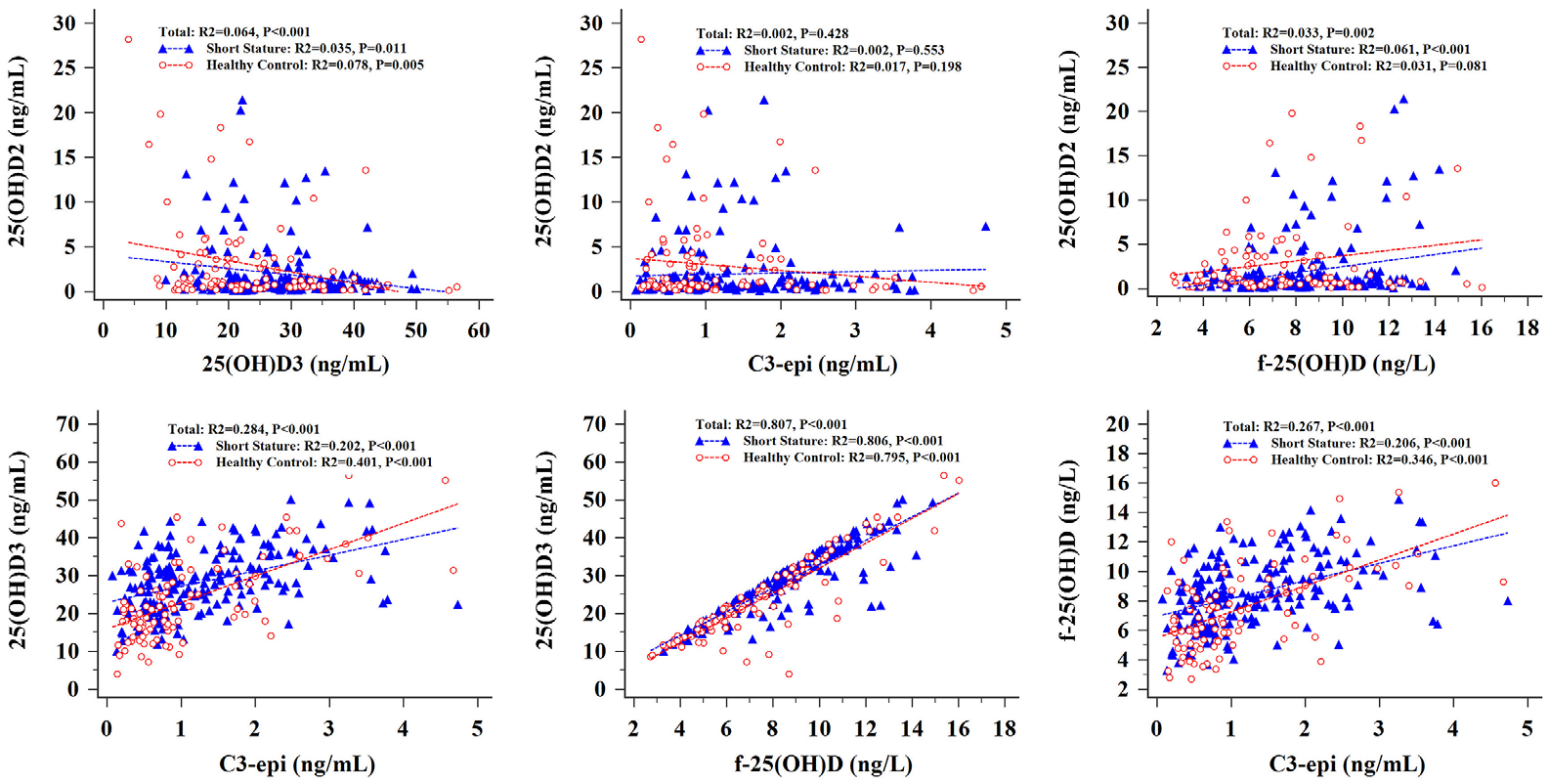
Correlation analysis of various vitamin D components in short stature and healthy control groups.

Analysis of the results shown in **Figure 2** revealed a weak negative correlation between 25(OH)D2 and 25(OH)D3 levels in short-stature and healthy control cohorts. Higher serum 25(OH)D3 concentrations were positively associated with higher values of C3-epi. However, there was no association between serum 25(OH)D2 levels and C3-epi concentrations. In addition, f-25(OH)D levels were positively correlated with 25(OH)D3 and C3-epi levels in both cohorts but were only positively connected with 25(OH)D2 in the short-stature group.

## Discussion

Several methods including radioimmunoassay, ELISA, and chemiluminescence have been utilized for determination of t-25(OH)D. However, they are incapable of separating 25(OH)D2, 25(OH)D3, and C3-epi from t-25(OH)D effectively (25). It has been reported that the potential of 25(OH)D3 in maintaining 25(OH)D levels is 2–3-fold better than 25(OH)D2, and the physiological importance of the C3-epi is not as yet very well known. Therefore, even the same values of t-25(OH)D may play differing physiological roles because of the different compositions of vitamin D components. LC-MS/MS can detect 25(OH)D2 and 25(OH)D3 levels simultaneously and distinguish C3-epi from 25(OH)D3. Thus, it is considered the “gold standard” method for measurement of vitamin D status. In the present study, LC-MS/MS was used to determine the serum levels of 25(OH)D2 and 25(OH)D3 and C3-epi levels in children with short stature, thereby accurately providing more information about the nutritional status of this disease.

Several previous studies have reported inverse associations between 25(OH)D concentrations and PTH levels in humans, but these findings were commonly observed in adults and old people (11). PTH levels in children included in the current study did not show dramatic elevations when serum 25(OH)D decreased significantly. This observation was consistent with findings of a previous report (26). This may be explained by the possibility that different mechanisms regulate the secretion of PTH during childhood and adolescence unlike in adults (27). Moreover, it is likely that 1,25(OH)2D, but not 25(OH)D, can directly influence PTH secretion (27, 28) and modulate the balance in calcium/phosphate and bone health by binding to the VDR (18). While the proportion of 25(OH)D that was converted into 1,25(OH)2D was uncertain in the current study, the clear causal association between PTH and 25(OH)D could not be explored. Meanwhile, the parathyroid cells also express 1-α hydroxylase (CYP27B1) so that they can produce their own active vitamin D in an autocrine fashion to regulate PTH production (28). The interaction of circulating and locally produced active vitamin D in the regulation of PTH synthesis is not entirely clear. In light of this, large-scale multicenter studies are needed to determine the association of vitamin D and PTH with short stature in children and provide ideas for developing accurate diagnostic tools and treatments for short stature.

Our results indicated that serum 25(OH)D3 levels in short-stature patients aged 7–12 and 13–18 years were lower compared with healthy participants during the same periods. Conversely, C3-epi levels and ratios of C3-epi/25(OH)D3 in all age ranges of short-stature children were higher than those in healthy subjects. C3-epi is the isomeric form of vitamin D3. Its active form, 3-epi-1α,25(OH)2D3, appears to have reduced calcemic effects than non-epimeric forms and can activate bone gamma-carboxy glutamic acid-containing protein (BGLAP, also called osteocalcin) at a much lower rate compared with 1α,25(OH)2D3 (29–31). However, there is still no clear causal association between C3-epi and short stature. Thus, the potential influences of C3-epi levels on height demand further elucidation. Accumulation evidence shows that the ratio of C3-epi/25(OH)D3 may be a promising tool to predict the status of various diseases such as Alzheimer’s disease, rheumatoid arthritis, and type 1 diabetes (32). The ratio of C3-epi/25(OH)D3 in the current study performed statistically different in short-stature and healthy children, indicating that it might also be a novel biomarker for vitamin D catabolism in children with short stature. It is important to estimate the percentage contribution of C3-epi to 25(OH)D3 across 25(OH)D3 concentration ranges, age ranges, and varying healthy statuses, which would enable the evaluation of the physiological processes of C3-epi.

The current study showed a weak positive correlation between C3-epi and 25(OH)D3 values in both short-stature and healthy children, which was consistent with previous studies on adults. However, this relationship could not hold in infant populations because the relative C3-epimer concentration is high in neonates and declines rapidly across infancy (29). Some studies postulated that increasing serum 25(OH)D3 concentrations switch on or activate putative epimerization enzyme (15). This may be a protective mechanism against excessive levels (48–56 ng/ml) and possibly unwanted influences of vitamin because the epimeric form may be less active compared with the non-epimeric form (15, 29). However, this process is likely to become saturated when 25(OH)D3 levels reach maximum (15). Therefore, the relationship between C3-epi and 25(OH)D3 may not always be linear. The current study indicated a more linear relationship between C3-epi and 25(OH)D3 values, probably due to the limited number of study subjects with too high serum 25(OH)D3 levels. Moreover, the correlation between 25(OH)D2 and C3-epi was explored in this study. No association between the two indicators was observed, revealing that vitamin D2 may not be a source of C3-epi. However, their relationships need further confirmation.

Approximately 0.03% of total 25(OH)D and 0.4% of total 1,25(OH)_2_D are free in circulation in healthy non-pregnant subjects. Its capacity depends on its physiological effects and body demands for vitamin D, rather than complex individual influencing factors (33–35). f-25(OH)D can freely move across membranes of kidney proximal tubule epithelial cells and be hydroxylated, indicating that it can be utilized by the body whenever needed (36, 37). Sufficient data from previous studies support speculation that free hormones (including free vitamin D) are more physiologically related compared with their total concentrations (38). Several scholars have argued that better skeletal conditions of African Americans despite their lower vitamin D levels (the African paradox) are likely due to the use of a “wrong” serum marker [t-25(OH)D], when f-25(OH)D should be the preferred indicator (39). Lower values of f-25(OH)D in short-stature children in age ranges of 7–12 and 13–18 years were observed in the current study, suggesting that available vitamin D levels decreased in short-stature young patients and f-25(OH)D is an alternative useful indicator for assessing vitamin D status in short-stature children. However, since several medical laboratories are incapable of determining f-25(OH)D values, assessment of the nutritional status of vitamin D in clinical practice is still challenging.

Total 25(OH)D comprising 25(OH)D2 and 25(OH)D3 is recommended by guidelines as the best indicator of vitamin D storage (40). Vitamin D3 levels are much higher compared with vitamin D2 levels, and vitamin D2/vitamin D3 ratios are extremely low in normal physiological conditions. Excessive 25(OH)D2 levels accompanied by significantly reduced 25(OH)D3 levels due to some unknown reasons may erroneously be interpreted as sufficient storage of vitamin D. Here, we found that after conversion of 25(OH)D2 to 25(OH)D3 activity (when vitamin D nutritional status is evaluated at the level of 25(OH)D3 activity equivalents), median vitamin D levels are decreased regardless of whether they are healthy or not. This may explain why serum vitamin D components and 25(OH)D2/25(OH)D3 ratios need to be determined. Findings of the current study showed that the 25(OH)D2/25(OH)D3 ratio in short stature was higher compared with that in control groups during age ranges of 7–12 and 13–18 years, indicating poor storage proportion of vitamin D3 in short-stature children.

C3-epi currently accounts for a significant proportion in neonates (21), infants, and even adults (41). The presence of C3-epi complicates the interpretation of serum 25(OH)D levels (42, 43). Otherwise, the capacity of vitamin D3 to store vitamin D is two to three times higher compared with that of vitamin D2. Therefore, three different methods were applied in the current study to determine vitamin D storage in short-stature children. Findings were totally varied, although it was clear that detection of t-25(OH)D [25(OH)D2+25(OH)D3] alone may overestimate vitamin D storage in short-stature children. Some previous studies had shown that epimeric interference does not significantly influence routine vitamin D determination for healthy adults using LC-MS/MS methods (44). This may be due to relatively low concentrations of C3-epimer in adults. However, due to lack of a more comprehensive understanding of the role of C3-epimer, determination of both 25(OH)D3 and C3-epimer in patients (especially infant and pediatric subjects) should be considered so that more accurate conclusions regarding the function of C3-epimer will be drawn with continued biological and molecular investigation.

Nevertheless, the current study had some limitations. First, it was limited by its retrospective nature with single academic center and relatively small sample size. Second, data on use of vitamin D supplements by participants were not collected. Similarly, information on sensitivity to sunlight, latitude, season, time of day, and how much direct sunlight that skin is exposed to was not included, all of which could be related to vitamin D status. In addition, methods for determining vitamin D metabolites were not standardized. High sensitivity of LC-MS/MS and poor reproducibility of ELISA may have led to certain variations in the obtained findings. Although reliability of the current study was not entirely satisfactory, it provides important reference for design and implementation of related studies.

## Conclusions

The current study revealed essential differences between various vitamin D contents in short-stature children compared with healthy ones. The findings indicated that short-stature patients had lower levels of vitamin D storage compared with healthy subjects. To accurately assess vitamin D nutritional status, kinds of vitamin D components in circulation including 25(OH)D2, 25(OH)D3, f-25(OH)D, t-25(OH)D, and C3-epi and ratios of C3-epi/25(OH)D3 and 25(OH)D2/25(OH)D3 should be determined extensively, in order to provide a scientific evidence-based basis for the diagnosis and treatment evaluation of short-stature individuals.

## Data Availability Statement

Datasets analyzed during the current study are available from corresponding author on reasonable request.

## Ethics Statement

The current study was approved by Medical Ethics Committee of Mianyang Central Hospital (approval no. P2020040). Written informed consent to participate in this study was provided by legal guardian/next of kin of participants.

## Author Contributions

All authors contributed to the current study conception and design and take responsibility for the integrity of data and accuracy of data analyses. Data collection was undertaken by BX, LG, and YZ, and analysis was undertaken by YF. Material preparation was done by and the first draft of the manuscript written by WJ, JF, and LY, and all authors commented on the previous versions of manuscript. All authors contributed to the article and approved the submitted version.

## Funding

The current work was financially supported by the Sichuan Health and Health Committee Support Program (20PJ255) and the Incubation Project of Mianyang Central Hospital (2019FH01 and 2019YJ22).

## Conflict of Interest

The authors declare that the research was conducted in the absence of any commercial or financial relationships that could be construed as a potential conflict of interest.

## Publisher’s Note

All claims expressed in this article are solely those of the authors and do not necessarily represent those of their affiliated organizations, or those of the publisher, the editors and the reviewers. Any product that may be evaluated in this article, or claim that may be made by its manufacturer, is not guaranteed or endorsed by the publisher.
